# Embedding chiropractic in Indigenous Health Care Organisations: applying the normalisation process model

**DOI:** 10.1186/1472-6963-12-429

**Published:** 2012-11-26

**Authors:** Barbara I Polus, Charlotte Paterson, Joan van Rotterdam, Dein Vindigni

**Affiliations:** 1 School of Health Sciences, RMIT University, Bundoora, 3083, Australia; 2 School of Social and Community Medicine, University of Bristol, Bristol, BS8 1TH, UK; 3 The University of Newcastle, Newcastle, 2308, Australia

## Abstract

**Background:**

Improving the health of Indigenous Australians remains a major challenge. A chiropractic service was established to evaluate this treatment option for musculoskeletal illness in rural Indigenous communities, based on the philosophy of keeping the community involved in all the phases of development, implementation, and evaluation. The development and integration of this service has experienced many difficulties with referrals, funding and building sustainability. Evaluation of the program was a key aspect of its implementation, requiring an appropriate process to identify specific problems and formulate solutions to improve the service.

**Methods:**

We used the normalisation process model (May 2006) to order the data collected in consultation meetings and to inform our strategy and actions. The normalisation process model provided us with a structure for organising consultation meeting data and helped prioritise tasks. Our data was analysed as it applied to each dimension of the model, noting aspects that the model did not encompass. During this process we reworded the dimensions into more everyday terminology. The final analysis focused on to what extent the model helped us to prioritise and systematise our tasks and plans.

**Results:**

We used the model to consider ways to promote the chiropractic service, to enhance relationships and interactions between clinicians and procedures within the health service, and to avoid disruption of the existing service. We identified ways in which chiropractors can become trusted team members who have acceptable and recognised knowledge and skills. We also developed strategies that should result in chiropractic practitioners finding a place within a complex occupational web, by being seen as similar to well-known occupations such as physiotherapy. Interestingly, one dimension identified by our data, which we have labelled ‘emancipatory’, was absent from the model.

**Conclusions:**

The normalisation process model has resulted in a number of new insights and questions. We have now established thriving weekly chiropractic clinics staffed by a team of volunteer chiropractors. We identified an ‘emancipatory’ dimension that requires further study. We provide a worked example of using this model to establish, integrate and evaluate a chiropractic service in an Indigenous Australian community.

## Background

Indigenous Australians continue to lag behind on all health indicators: life expectancy is 15–20 years less than non-Indigenous Australians and rates of hospitalisation, poor health and quality-of-life show similar disparities [1]. Factors such as distance from services, availability of culturally appropriate services, workforce shortages and private health insurance cover all affect access to, and the utilisation of, health services [1]. Aboriginal Community Controlled Health Services are the preferred and most culturally appropriate organisations to deliver health services to Indigenous people [2]. In addition to the normal range of clinical staff, they employ Aboriginal Health Workers (AHWs) who have the trust, respect and local knowledge required in promoting the health of their community through health screening and the assessment of diseases including diabetes and mental health [3,4]. Against this backdrop of unmet need, a not-for-profit organisation, Hands on Health Australia, has worked for 15 years in a rural Indigenous community in New South Wales to firstly establish the prevalence and types of musculoskeletal pain and associated disability [3,4] and secondly to introduce services and training to better manage and prevent these conditions [5]. This involvement has, from the start, been based on the philosophy of keeping the community involved in all phases of development, implementation, and evaluation (http://www.ahmrc.org.au/). Research in a number of Aboriginal communities [6,7] indicates that chiropractic and massage may, in addition to treating musculoskeletal conditions, promote self-care in ways that improve general wellbeing and positively influence other chronic illnesses. These findings accord with research in marginalised communities in Canada, which indicates that chiropractors can function well in a collaborative environment with conventional care providers, and can also effectively contribute and participate in public health initiatives [8-10].

In 2004 a chiropractic service was established in a large Aboriginal community. It built on the previous years of research and extensive discussions with a community advisory group, including community leaders and medical and allied health practitioners [11]. It was staffed by volunteer chiropractors. During the first few years, fortnightly clinics were maintained at the Durri Aboriginal Medical Service (ACMS) and an additional chiropractic service was made available at the aged-care facility of the Booroongen Djugun Aboriginal Corporation. Community and clinician consultation meetings continued to be held, and an evaluation project was designed and funded. Phase 1 of the project aimed to evaluate the establishment and development of an ongoing chiropractic programme. The evaluation was based on a Participatory Action Research (PAR) approach [12] and was focused on establishing a sustainable, well used and high quality service. The aim of the second phase was to describe and measure the effects of chiropractic care in this community. Despite being located in one of Australia’s largest rural Aboriginal communities there were problems with securing sufficient referrals to the service and this was compounded by concerns about sustaining funding and practitioner availability. Through Phase 1 of the evaluation process, the difficulties facing the chiropractic service and the work that was needed to overcome them became more explicit, their complexities better understood and the need to rectify them more urgent.

In 2006 the eminent sociologist, Carl Mays, described the ‘normalisation process model’ that aims to understand the practical problems of embedding and evaluating new complex interventions [13]. His approach offered a framework for organising the many tasks that confronted us – tasks that spanned difficulties in referrals, funding and building sustainability as well as the evaluation itself. This model has since been developed into a middle-range theory [14], but we have found the model itself most useful for our purposes. There are many other models of integrative care that address how success can be achieved but they are generally reported in relation to evaluating established services rather than providing a structure for the very early development stages. For example, the excellent and widely used framework by Boon et al. (2004) will enable us to describe and evaluate our service, once it is better established, in terms of four key components of integrative health care practice - philosophy/values; structure, process and outcomes [15]. Another framework of particular interest – that of Mior and colleagues for the integration of chiropractic services into a multidisciplinary practice in Canada [16] – was being developed in parallel with our own work and is discussed later.

This paper describes using the normalisation process model [13] to establish, integrate and evaluate a chiropractic service in a rural, Indigenous Australian community. In order to make it ‘fit for purpose’ in our particular context we made some adaptations, especially in relation to language and terminology. We demonstrate how an academic conceptual model can be interpreted and adapted for use in a complex practice situation.

## Methods

### The normalisation process model

The structure and original terminology of the model is provided in Table 1. The Normalisation Process Model is a tool that enables a “*practical understanding of the conditions in which complex interventions can become embedded within clinical work*” by “*understanding the practical problems of workability and integration that complex interventions pose*” [13]. In other words, “*how and why things become*, *or don*’*t become*, *routine and normal components of everyday work*” [17]. It acknowledges that healthcare workers and organisations value stability and order but are capable of adapting new interventions to meet specific *local* situations and requirements. It aims to develop a deeper and more general understanding of a situation rather than, for example, describing it in terms of observed barriers and facilitators to change. The model was derived from extensive and robust secondary analysis and synthesis of numerous qualitative research studies investigating chronic disease management in primary care in the UK. The model, and the theory which it generated, have since been used by other researchers including, for example, a team seeking to embed effective depression care within Australian primary care [18]. We used the following, somewhat overlapping, methodological steps to adapt the terminology of the dimensions and to apply them to our own data. Consensus decision making was used throughout the process [19].

a. The original model and related literature were studied and discussed by the authors of this paper and were considered in relation to their experiences with the chiropractic service.

b. Previous meeting notes, action research data, and relevant email discussions from the previous three years were systematically coded into the model dimensions. This was done independently by two of the authors (BP & CP) and commented on by others.

c. Matrices were used to display this evolving categorisation of our data and to explore the utility of new terminology. These highlighted potential ‘empty categories’ and areas of uncertainty or ambiguity. These were discussed.

d. During this process the terminology of the original model was critiqued and more everyday wording was developed and applied. The original terminology was retained alongside, to ensure the concepts remained as grounded and detailed as possible.

e. As new data were collected these were added into the emergent model and ‘deviant case analysis’ was used to promote discussions of data that did not fit the model.

**Table 1:** The Normalisation Process Model-a summary of the original model (adapted from May 2006)

**Endogenous Processes**		
**Endogenous processes comprise elements of professional/patient relations and their associated material practices in the clinical encounter**		
**Construct, Dimension and Proposition**	**Dimension**	**Components**
***1: Interactional workability***	*1.1 Congruence*	Co-operation
The interactional work that professionals and patients do within the clinical encounter and its temporal order	(the process of interaction)	(shared expectations, minimise disruption)
		Legitimacy
		(shared beliefs about objects and roles)
		Conduct
		(verbal and non-verbal)
	*1.2 Disposal*	goals
	(the effects of interaction)	meaning
		outcomes
Proposition 1: A complex intervention is disposed to normalization if it equals or improves accountability and confidence within networks.		
***2: Relational integration***	*2.1 Accountability*	Validity
The embeddedness of trust in professional knowledge and practice	(internal credibility)	Expertise
		Dispersal
	*2.2 Confidence*	Credibility
	(external credibility)	Utility
		Authority
Proposition 2: A complex intervention is disposed to normalization if it equals or improves accountability and confidence within networks.		
**To summarize (constructs 1 & 2): The clinical encounter and the social relations that surround it are historically and culturally stable. Where a complex intervention interferes with the order of professional/patient interaction, either by disrupting the interaction between professionals and patients, or by undermining confidence in the knowledge and practice that underpins it, then it is also an unlikely candidate for normalization.**		
**Exogenous processes**		
Exogenous processes comprise the ways that work is organized, its division of labour, and the institutional structures and organizational processes in which it is located		
***3: Skill set workability***	*3.1 Allocation*	Distribution
The organizational distribution of work, knowledge and practice across divisions of labour		Definition
		Surveillance
	*3.2 Performance*	Resourcing
		Power
		Evaluation
Proposition 3: A complex intervention is disposed to normalization if it is calibrated to an agreed skill-set at a recognizable location in the division of labour.		
**4: Contextual Integration**	4.1 Execution	Resourcing
The capacity of the health care organization to allocate control and infrastructure resources and to negotiate integration into existing activities	(the ownership of control over the resources and agents required to implement chiropractic)	Power
		Evaluation
	*4.2 Realization*	Risk
	(the allocation and ownership of responsibility for implantation)	Action
		Value
Proposition 4: A complex intervention is disposed to normalization if it confers an advantage on an organization in flexibly execution and realizing work.		
**To summarise (constructs 3 & 4): to be an optimal candidate for normalization, a complex intervention must “fit” with an actual or realizable set of roles within an organizational or professional division of labour, and at the same time must be capable of integration within existing or realizable patterns of service organization and delivery.**		

## Results

In this section we describe how the insights we gained from the model helped us to embed chiropractic in our particular context. We present our own interpretation and application of the model using simpler terminology (original model terminology is placed in brackets). We briefly describe each component of the model and the associated actions and plans. The model has two major categories - A: endogenous processes and B: exogenous processes. Within each of these categories there are two constructs and each construct is described in terms of two dimensions (Table 1).

### Chiropractic in relation to consultations between patients and doctors and other health workers (endogenous processes)

#### Promoting good relationships between patients and doctors/health workers

The first construct of the model emphasises the importance of avoiding disruption of the patient-practitioner relationship (interactional workability). Instead, we should try and enhance the process and outcome of relevant consultations (e.g. for back pain).

##### Dimension 1.1

*This is firstly considered in terms of trying to promote shared expectations and beliefs about chiropractic so that consultations are interactionally* ‘*comfortable*’ *for both parties and of a normal length* (*Congruence*).

Bridging the gap between lay and medical expectations and beliefs about chiropractic is a difficult task, especially in the national context of considerable negativity between the medical profession and chiropractic. Many health workers and staff indicated to us that they know little about chiropractic or how it might help their patients or residents and that they would welcome information and discussion. Consequently, the project chiropractor and one of the authors, herself a chiropractor (JvR) have started making presentations at routine staff meetings and staff are encouraged to attend the clinic for treatment for their own musculoskeletal problems. We also tackled this issue by working more closely with AHWs, who generally have a positive attitude towards holistic physical therapies. We have also commenced development of clinical pathways for residents of the Aged Care facility who required informed consent from their relatives. Another approach to promoting shared aims was to focus on the aims of patients and community leaders that are likely to resonate with GP concerns. For example, as part of the Participatory Action Research a wide range of stakeholders developed affinity diagrams and the commonest theme for responses to a question about the aims of the chiropractic service was: ‘*For the Aboriginal Corporation Medical Service to be a recognised place that the community can go to get pain relief without medications*’. Future fact sheets and presentations will highlight the role that chiropractic could play in addressing shared concerns such as the importance of promoting mobility and exercise, the desire to avoid referral for surgery and/or medication (especially when medication load is already high), and long waiting lists for physiotherapy. However, community consultations also produced the following suggestion that appears not to fit into the model. A recurrent theme in meetings was that because some doctor’s views were ‘*very entrenched*’ they may best be influenced by ‘*patient demand*’. Consequently raising ‘*community awareness*’ was considered an important strategy. However, as it is unusual for patients to initiate preferences for a new treatment option in medical consultations, promoting this for chiropractic would be likely to threaten the doctor-patient relationship. Consequently the model has led us to suggest that in addition to raising community awareness, patients should be encouraged to discuss referral with AHWs, who see themselves as advocates for patients. This was also the preferred approach of the AHWs who attended or had input to the community consultations.

##### Dimension 1.2

*Another potential disruption of the patient*-*clinician relationship is lack of agreement about what the outcome of the consultation will be* (*Disposal*). *This could include what the clinician may do* (*refer or not to the chiropractor*) *what the outcome of seeing a chiropractor might be* (*degree and duration of improvement*, *safety issues*) *and what effect seeing the chiropractor may have on the doctors workload* (*issuing of sick notes*, *medication*).

All of these issues were raised in the consultation meetings. For example an AHW specifically asked about medical certification and said that knowing that chiropractors could provide certificates was important. Also discussions about claiming Enhanced Primary Care (EPC) payments (additional Medicare funding that can be claimed for specified allied health services) were helped by sharing information about the chiropractor already having his own provider number for this scheme, thereby simplifying the claim procedures. This aspect of the model encouraged us to find ways of ensuring that appropriate referrals for chiropractic are a popular and easily agreed outcome for a consultation. Most importantly we created appropriate referral pathways between chiropractors and the GPs so that chiropractic services are covered through the Enhanced Primary Care (EPC) scheme. This system includes actions by chiropractors as well as referrals through the AHWs, and we are also working with the director at the Aged-Care facility of Booroongen Djugun to improve the uptake of EPC referrals for the provision of allied health services to residents. In addition to making referral easier within consultations, payments from this scheme will assist in sustainable funding of the chiropractic service. As Phase 2 of our evaluation proceeds, the findings will provide more detailed and context specific information on process and outcomes for clinicians and patients.

#### Health team confidence on the knowledge base and expertise of chiropractic

The second construct of the model concerns the need for chiropractic to fit into the known and trusted assumptions of the immediate network surrounding individual consultations (relational integration).

##### Dimension 2.1

*The model suggests that trust is built on relative agreement about theknowledge baseand the expertise required for any intervention*. *Thus it requires some shared beliefs about what chiropractors know*, *whether that knowledge is sound*, *how they act on it and how aspects of it can be shared* (*Accountability*).

##### Dimension 2.2

*Attempts should also be made to promote shared understanding and beliefs* – *or at least not undermine them* - *about whatsources of knowledgeare seen as credible*, *useful and authoritative in the clinical work being engaged with and what criteria should be used toassess knowledge* (*Confidence*).

At a meeting with GPs a number of misunderstandings about chiropractic were discussed. GPs requested evidence for the effectiveness of manipulation and raised questions and concerns about the mechanisms and likely effects of chiropractic care in certain conditions. We compiled and circulated a summary of the research evidence for the effectiveness and safety of chiropractic and although it has proved difficult to arrange further meetings with clinicians, we continue to give it priority. Building confidence in the knowledge base of chiropractic is likely to take time and to accompany increased interaction with chiropractors and experiences of treatment by staff and patients. Chiropractors are encouraged to describe the extent of their training and the increasing numbers of national clinical guidelines that include manipulative therapies as a treatment option.

### Chiropractic in relation to the whole organisation and the professional and occupational roles and responsibilities that constitute its work (exogenous processes)

#### Slotting in to an occupational niche and team position

The third construct of the model reminds us that new interventions and practitioners need to fit into actual or potential occupational roles (skill-set workability) in the organisation.

##### Dimension 3.1

*Each healthcare organisation will have formal and informal policies about who* (*doctor*, *nurse*, *clerical staff etc*.) *does which tasks*, *how they work together to care for different groups of patients*, *and how status and rewards are allocated*. *There are also agreements about who has what skills or groups of skills and how the application of those skills is assessed or audited* (*Allocation*).

This was a dimension that was often raised in our consultations with staff and several specific suggestions were made. Several staff believed that it was important that chiropractors were properly paid and valued and were given appropriate rooms to work in. It became apparent to the chiropractors they could fit into the organisation better if they promoted themselves as being a similar occupational group to, for example, physiotherapists. They also realised that they could use the team working methods employed by physiotherapists; for example by initiating feedback and cross-referral by email to other members of the chronic care team. The physiotherapist who worked in the Medical Centre has supported the chiropractic clinic from the outset which makes this suggestion especially valuable. Recently we have been working with the clinical practice coordinator at the medical centre to ensure that all volunteer chiropractors have access to and utilise the electronic clinical database to record their clinical notes. Use of this database necessitates chiropractors coding their examination, diagnosis and treatment into recognised fields within the database (e.g. musculoskeletal pain of the spine). This access to the internal clinical records database for notes of patients will also enable email communication and cross-referral. This electronic database is not available at the Aged-Care facility, but the research team is working with the administration to establish a patient clinical record system for the chiropractors that includes the recording of informed consent for treatment procedures, accurate patient history, examination findings and treatment provided. A secure filing cabinet has been provided and strategies are being developed to link clinical records of the facility and the chiropractic clinic so that co-management of patients is facilitated.

###### Dimension 3.2

*Another requirement for embedding a new service into the organisation is the capacity of the workforce to slot it into the most appropriate place in the menu of services available*. *This will require shared understandings about competencies*, *autonomy and quality assurance issues*, *especially in areas where there is potential overlap with established work teams*. (*Performance*)

This dimension was reflected in a comment by an AHW who said that the chiropractic programme had to ‘develop its own identity and sell itself’ and that in the short term it needed nurturing by specific people who supported it. Members of the research team have addressed this issue by a focus on increasing the numbers of volunteer chiropractors and hence the frequency of the clinics. We have now achieved a team of volunteer chiropractors and weekly clinics at both sites, thus providing a presence that has the potential to develop an identity and establish a role within the teams that provide services in the centres. The ability to provide weekly treatment for individual patients will also improve the quality of the service being provided. During the same period we have developed an on-going relationship, with the clinical practice, between the chiropractors and other clinicians.

#### Integrating into the structure and resources of the organisation and wider context

The final section of the model concerns the structural and resource implications of integrating the new intervention (contextual integration).

##### Dimension 4.1

*Who has the power*? *We need to understand who allocates each of the resources that the chiropractic service may require and what formal and informal policies are used to minimise disputes between different programmes of work about these resources*, *costs*, *and evaluation procedures* (*Execution*).

##### Dimension 4.2

*Who is going to do the work of integration*? *Implementing the new service will require clear lines of responsibilities for making the change in such a way that potential risks are defined and managed and resources are negotiated*. *Realising this change will be easier if expectations about the value of the new service are realistic and explicit*.

This dimension has been an important one at times, when a series of changes in personnel in management positions at the ACMS inevitably slowed the progress of our project. During this period we remained aligned and committed to Aboriginal ownership of the project and sought their advice and support. After a difficult period, we have once again had endorsement of the project by the CEO of the ACMS and renewed opportunities to work closely with the management team. At the same time we formally sought permission for the research from the new Director of Care of the aged-care facility, whose attitude to the chiropractic programme dramatically changed once this was done and she understood the history of the programme. The chiropractic service was seen to fit with the mission of the Aboriginal Corporation owned aged-care facility, which was ‘to improve the life of the residents’. The outcome of all this preparatory work meant that during our consultations in February 2011 we were in a position to draw up a protocol for introducing new programmes into Indigenous communities:

1. Consultation must start with the Elders of the Indigenous community and this process needs to be at the highest level with the entire research team. At this initial meeting the projected advantages of the new program to the community should be outlined, as well as the role of the community consultation process that will include the community in the decision making process. The research project that will evaluate the process should be explained and how the particular project will be used to build a sustainable programme.

2. Consultation with relevant CEOs outlining the same as above.

3. Consultation with the clinical practice co-ordinator and medical and allied health staff to outline the programme and the research process; this is a matter of professional courtesy.

4. Consultation with relevant practice managers to determine the practical implementation of the programme.

5. Consultation with office staff regarding the practical implementation of the programme.

These consultations are not one-off affairs and our meetings will be structured to apply the model in our particular situation. The self-funding nature of the chiropractic service has meant that we have not required financial resources from the health care organisation but the provision of space and the hosting of the evaluation have required ongoing negotiations and sometimes some delays. Although disquiet about the potential risks of manipulation was not openly discussed it is likely to be important and requires more exploration. Providing the organisation with certificates of professional insurance and developing protocols for risk reduction and management may be helpful.

## Discussion

The concept of ‘normalisation’ or ‘embedding’ a new service focuses on the importance of sustainability. The Normalisation Process Model emphasises the importance of stability and order in health care, both within one-to-one consultations and within the organisation as a whole. In the context of an Australian Aboriginal community it directed us to consider ways to promote chiropractic as something that will enhance relationships, interactions and procedures, and avoid disrupting them. This should be achievable, given that chiropractic requires few resources and offers an alternative referral option for conditions that are difficult to treat within biomedicine. The model also emphasises the importance of chiropractors becoming trusted team members who have acceptable and recognised knowledge and skills; this is more of a challenge given the national Australian context in which chiropractors are still marginalised in healthcare organisations. Our results suggest that chiropractors should be able to find a place within a complex occupational web, by being seen to be very similar to well known occupational groups such as physiotherapists. We have been able to improve the potential for team working by providing more frequent clinics, by negotiating access to electronic and paper clinical systems and by linking into established and referral systems. The importance of the organisational context has become apparent as we try to establish an evaluation project. The model’s section on identifying who has the power, as well as who does the work, may help us to find our way through the complexity of the health care organisations. In our early consultations, members of the community emphasised the need to raise community awareness about the chiropractic service, but insights from the model suggest that arranging more focused meetings with health workers to discuss team working and communication are also important.

Our consultations and experience highlight the existence of a theme that is not identified by the model, which may be described as emancipatory. The verb ‘emancipate’ is defined as ‘set free, especially from legal, social or political restrictions’ [20]. The normalisation model conceptualises an intervention as becoming embedded through aligning itself with the status quo and current biomedical beliefs, values etc., rather than any suggestion that the intervention could be demanded by patients and introduced in order to change the status quo. Whilst this reflects the body of UK research that May synthesised, it may need amending for use in the context of an Indigenous culture struggling with a lack of culturally sensitive healthcare. For example, a strong sense from the community members was “*the importance of the tactile therapies in healing their people in not only physical ways but healing emotional injuries as well* (*the anger in the communities*)”. Community and staff members also used participatory action research methods and individually specified aims for the chiropractic service. There was a wide range of individual aims but the one that best sums up the commonest themes is “*For the ACMS to be a recognised place that community can go to get pain relief without medications*”. Other common themes were a desire for a holistic approach, for community wellness and empowerment, and for better acceptance and communication of chiropractic. The belief that informing patients about the service would lead to doctors referring to the service because of patient demand was strongly held. As it stands the model suggests this would not work, because both patient and doctor wish to avoid difficult interactions which threaten their relationship. It remains to be seen whether Indigenous structures and community support, and the advocacy of the AHWs, will allow patients to be more assertive in their interactions with doctors. Ongoing discussions with the Indigenous community, plus the results of our mixed method evaluation, may help us to define more clearly whether an emancipatory dimension is an important addition to the normalisation process model in this context.

Our findings accord with other evaluations of integrating chiropractic care into mainstream services. For example the key success factors identified by Kopansky-Giles et al. (2010) were: the importance of champions, laying groundwork, the organisational culture and the choice of practitioners; and the barriers in this inner-city setting were: funding, lack of awareness of the service and perceptions of risk [21]. The importance of champions and of trust in the ‘right’ practitioners has been confirmed elsewhere [22] and are issues that continue to be central to our project as we move into the next phase. In terms of the specific aim of embedding chiropractic into marginalised communities, future work will also include comparing our approach with that of Mior and colleagues who recently developed a framework for the integration of chiropractic services into a multidisciplinary practice in Canada [16]. As the key categories of that framework are communication, practice parameters and service delivery, it would appear that our two approaches are complementary and that further work within the context of health care for First Nation People will further strengthen the link between theory and practice. Aboriginal populations in Australia, New Zealand, Canada and the USA share many commonalities and a number of initiatives have demonstrated that well-resourced, community-controlled and culturally appropriate and accessible programs can, and do, have a positive impact, and result in significant and sustained improvement in the health outcomes of Aboriginal people [23].

The normalisation process model and theory has been successfully used in other contexts, such as implementing change in primary care for depression [18], evaluating a self-management training package in primary care [24], and normalizing a new technology in infertility management [25]. However, the application of the theory that these papers describe is complex and thus likely to be inaccessible to many non-academic service providers. The model with a revised terminology described in this paper, and the example of its application in a marginalised community, may be of great benefit to others seeking to embed new services into mainstream healthcare. The limitations of our study include the use of a single case study and the emergent nature of our understanding of the theory, which were compounded by the remoteness of the community and our limited resources. However, the utility of our adapted model in the face of these difficulties indicates that it may be applicable in many other poorly resourced settings. The authors of the normalisation process theory, acknowledging the need to translate it to make it useful for non-academic health service providers, have recently developed a simplified web-based version [26]. In this respect, this paper is part of an important movement to translate the theory from its abstract form to one that can be used to solve problems in everyday settings.

## Conclusions

We have adapted the language and terminology of the normalisation process model to make it ‘fit for purpose’ in our community context. This adapted model has provided us with a structure for organising the data from consultation meetings and prioritising tasks, as well as providing us with a number of new insights and questions. It has led us to focus on ways in which chiropractic can enhance relationships, interactions and procedures and avoid disrupting them and also the importance of chiropractors becoming trusted team members who have acceptable and recognised knowledge and skills. Our experience of using the model in the context of an Indigenous community suggests that an additional ‘emancipatory’ dimension may be required.

## Competing interests

The authors declare that they have no competing interests.

## Authors’ contributions

BP, DV, JvR developed the programme of research, the chiropractic service and the consultation meetings and helped to draft the paper. CP conceived of and drafted the main outline of the paper and participated in designing and consulting on the service evaluation. All authors read and approved the final manuscript.

## Pre-publication history

The pre-publication history for this paper can be accessed here:

http://www.biomedcentral.com/1472-6963/12/429/prepub

## References

[B1] Australia's Health The Twelth Biennial health report of the Australian Institute of Health and Welfare 2010 Canberra: Australian Government, Australian Institute of Health and Welfare

[B2] Pacza T Steele L Tennant M Rural and remote oral health, problems and models for improvement: a Western Australian perspective Aust J Rural Health 2000 34 822 28 10.1046/j.1440-1584.2000.81224.x 11040576

[B3] Vindigni D Griffen D Parkinson J Da Costa C Parkinson L The prevalence of musculoskeletal conditions, associated pain and disability and the barriers to managing these conditions in a rural, Australian Aboriginal Community Rural Remote Heal 2004 4 no 230. Available from http://www.rrh.org.au/articles/showarticlenew.asp?ArticleID=230 15885010

[B4] Vindigni D Parkinson L Rivett D Da Costa C Perkins J Walker BF Blunden S Developing a musculoskeletal screening survey for Indigenous Australians living in rural communities Rural Remote Heal 2006 6 321 (Online). Available from http://www.rrh.org.au/articles/showarticlenew.asp?ArticleID=321 16566661

[B5] Vindigni D Parkinson L Walker B Blunden S Rivett D Perkins J 'Hands-on Aboriginal Health: A Community-based sports massage course for Aboriginal Health Workers' Aust J Rural Health 2005 13 111 115 10.1111/j.1440-1854.2005.00664.x 15804336

[B6] Vindigni D Walker BF Jamison JR Da Costa C Parkinson L Low back pain risk factors in a large rural Australian Aboriginal Community. An opportunity for managing comorbidities. Chiropractic and Osteopathy Chiropr Osteopat 2005 13 21 10.1186/1746-1340-13-21 16197555 PMC1277832

[B7] Paterson C Vindigni D Polus B Browell T Edgecombe G Evaluating a massage therapy training and treatment programme in a remote Aboriginal community: methods and preliminary findings Complement Ther Clin Pract 2008 14 158 167 10.1016/j.ctcp.2008.03.004 18640627

[B8] Kopansky-Giles D Vernon H Steiman I Tibbles A Decina P Goldin J Kelly M Collaborative community-based teaching clinics at the Canadian Memorial Chiropractic College: addressing the needs of local poor communities J Manipulative Physiol Ther 2007 80 558 565 17996546 10.1016/j.jmpt.2007.06.008

[B9] Johnson C Baird R Dougherty PE Globe G Green BN Haneline M Hawk C Injeyan HS Killinger L Kopansky-Giles D Chiropractic and public health: current state and future vision J Manipulative Physiol Ther 2008 31 397 410 10.1016/j.jmpt.2008.07.001 18722194

[B10] Garner MJ Birmingham M Aker P Moher D Balon J Keenan D Manga P Developing integrative primary healthcare delivery: adding a chiropractor to the team Explore 2008 4 18 24 10.1016/j.explore.2007.10.003 18194787

[B11] Vindigni D Polus B Edgecombe G Van Rotterdam J Turner N Spencer L Irvine G Walsh M Bringing Chiropractic to Aboriginal communities: The Durri model Chiropract J Aust 2009 39 80 83

[B12] Kemmis S McTaggart R The action research reader 1990 Victoria, Australia: Deacon University Press

[B13] May C A rational model for assessing and evaluating complex interventions in health care BMC Health Serv Res 2006 6 86 10.1186/1472-6963-6-86 16827928 PMC1534030

[B14] May CR Mair F Finch T MacFarlane A Dowrick C Treweek S Rapley T Ballini L Ong BN Rogers A Development of a theory of implementation and integration: Normalization Process Theory Implement Sci 2009 4 29 10.1186/1748-5908-4-29 19460163 PMC2693517

[B15] Boon H Verhoef M O’Hara D Findlay B From parallel practice to integrative health care: a conceptual framework BMC Health Serv Res 2004 4 15 10.1186/1472-6963-4-15 15230977 PMC459233

[B16] Mior S Barnsley J Boon H Ashbury FD Haig R Designing a framework for the delivery of collaborative musculoskeletal care involving chiropractors and physicians in community-based primary care J Interprof Care 2010 24 678 689 10.3109/13561821003608757 20441400

[B17] May C Finch T Implementing, Embedding, and Integrating Practices: An Outline of Normalization Process Theory Sociology 2011 43 535 10.1177/0038038509103208

[B18] Gunn J Palmer VJ Dowrick C Herrman HE Griffiths FE Kokanovic R Blashki GA Hegarty KL Johnson CL Potariadis M Embedding effective depression care: using theory for primary care organisational and systems change Implement Sci 2010 5 62 10.1186/1748-5908-5-62 20687962 PMC2925331

[B19] Hartnett T The basics of consensus decision makinghttp://www.consensusdecisionmaking.org (accessed 13.10.2012)

[B20] Stevenson A Oxford Dictionary of English 2010 3 Oxford: Oxford University Press

[B21] Kopansky-Giles D Vernon H Boon H Inclusion of a CAM therapy (chiropractic care) for the management of musculoskeletal pain in an integrative, inner city, hospital-based primary care setting J Alternative Med Res 2010 2 61 74

[B22] Boon HS Kachan N Integrative medicine: a tale of two clinics BMC Compl Alternative Med 2008 8 32 10.1186/1472-6882-8-32 PMC2443104 18564418

[B23] Freemantle J Officer K McAullay D Anderson I Australian Indigenous Health—Within an International Context 2007 Darwin, Australia: Cooperative Research Centre for Aboriginal Health http://aboriginal.childhealthresearch.org.au/media/57190/austindigneoushealthreport.pdf (accessed 13.10.12)

[B24] Kennedy A Chew-Graham C Blakeman T Bowen A Gardner C Protheroe J Rogers A Gask L Delivering the WISE (Whole Systems Informing Self-Management Engagement) training package in primary care: learning from formative evaluation Implement Sci 2010 5 7 10.1186/1748-5908-5-7 20181050 PMC2841580

[B25] Wilkes S Rubin G Process evaluation of infertility management in primary care: has open access HSG been normalized? Prim Health Care Res Dev 2009 10 290 298 10.1017/S1463423609990168

[B26] May CR Finch T Ballini L MacFarlane A Mair F Murray E Treweek S Evaluating complex interventions and health technologies using normalization process theory: development of a simplified approach and web enabled toolkit BMC Health Serv Res 2011 11 245 10.1186/1472-6963-11-245 21961827 PMC3205031

